# Gain-of-Function Mutations in* STAT1*: A Recently Defined Cause for Chronic Mucocutaneous Candidiasis Disease Mimicking Combined Immunodeficiencies

**DOI:** 10.1155/2017/2846928

**Published:** 2017-11-13

**Authors:** Sanem Eren Akarcan, Ezgi Ulusoy Severcan, Neslihan Edeer Karaca, Esra Isik, Guzide Aksu, Mélanie Migaud, Ferda Evin Gurkan, Elif Azarsiz, Anne Puel, Jean-Laurent Casanova, Necil Kutukculer

**Affiliations:** ^1^Ege University Medical Faculty, Department of Pediatric Immunology, Izmir, Turkey; ^2^Ege University Medical Faculty, Department of Pediatric Genetics, Izmir, Turkey; ^3^Laboratory of Human Genetics of Infectious Diseases, INSERM, Paris, France; ^4^Ege University Medical Faculty, Department of Pediatrics, Izmir, Turkey; ^5^St. Giles Laboratory of Human Genetics of Infectious Diseases, The Rockefeller University, New York, NY, USA

## Abstract

Chronic Mucocutaneous Candidiasis (CMC) is the chronic, recurrent, noninvasive Candida infections of the skin, mucous membranes, and nails. A 26-month-old girl was admitted with the complaints of recurrent oral Candidiasis, diarrhea, and respiratory infections.* Candida albicans* grew in oral mucosa swab. CMV and EBV DNA titers were elevated. She had hypergammaglobulinemia; IgE level, percentages of lymphocyte subgroups, and in vitro T-cell proliferation responses were normal. She had parenchymal nodules within the lungs and a calcific nodule in the liver. Chronic-recurrent infections with different pathogens leading to significant morbidity suggested combined immunodeficiency, CMC, or Mendelian susceptibility to mycobacterial diseases. Genetic analysis revealed a predefined heterozygous gain-of-function mutation (GOF) (c.1154 C>T, p.Thr385Met) in the gene coding STAT1 molecule. Hematopoietic stem cell transplantation (HSCT) was planned because of severe recurring infections. Patients with* STAT1* GOF mutations may exhibit diverse phenotypes including infectious and noninfectious findings. HSCT should be considered as an early treatment option before permanent organ damage leading to morbidity and mortality develops. This case is presented to prompt clinicians to consider* STAT1* GOF mutations in the differential diagnosis of patients with chronic Candidiasis and recurrent infections with multiple organisms, since these mutations are responsible for nearly half of CMC cases reported.

## 1. Introduction

Chronic Mucocutaneous Candidiasis (CMC) is a general name used for the chronic or recurrent, noninvasive* Candida* infections of the skin, mucous membranes, and nails. Primary immune deficiencies (PID) should be considered after exclusion of secondary reasons affecting the immune system such as prolonged immunosuppressive drug use (glucocorticoids), infections (HIV), or metabolic defects (diabetes mellitus) [1].

CMC is frequently a component of combined immunodeficiencies (with decreased T-cell number or function) in which susceptibility to various infectious agents and noninfectious signs such as autoimmunity is expected. CMC may be the single or coexisting infectious condition in some other PID syndromes such as autosomal recessive (AR) autoimmune polyendocrinopathy syndrome type I (AR AIRE mutations), autosomal dominant (AD) hyper IgE syndrome (AD STAT3 mutations), AR caspase recruitment domain-containing protein 9 (CARD9) deficiency with invasive fungal diseases, AR IL12 receptor-beta1, and IL12-p40 deficiency causing susceptibility to mycobacterial diseases. There is also one more group of patients having CMC as the most prominent feature and defined as CMC disease (CMCD) [1–3].

Despite the narrow definition, most chronic mucocutaneous Candidiasis disease (CMCD) patients were reported to have further susceptibilities to noncandidal fungal and nonfungal infections and develop noninfectious findings such as autoimmunity, aneurysms, and cancer [1, 2].

Herein, we present a CMC patient with granulomas in the lungs and the liver, persistent oral Candidiasis, eosinophilic esophagitis, bronchiectasis, and recurrent diarrhea suggesting a combined immunodeficiency. The cause of the disease was found to be gain-of-function (GOF) mutation in transducer and activator of transcription 1* (STAT1)* which was recently reported to lead to CMCD; the literature about* STAT1* GOF mutations was also reviewed.

## 2. Case Report

A 26-month-old girl was admitted to inpatient clinics with the complaints of recurrent oral Candidiasis, lower respiratory tract infections, and diarrhea. Candidiasis recurred whenever antifungal treatment was interrupted since the newborn period. She had bronchiolitis, pneumonia, and otitis media recurring 4-5 times a year, and these infections had started in the first month of life. She had a severe varicella infection when she was 5 months old. She had mild mental and motor developmental delay. She was the second child of third-degree consanguineous healthy parents. Her elder six-month-old brother died due to pneumonia. No further information on the nature of his infection could be obtained, and it was learned that no evaluation was carried out for him.

**Figure 1:**
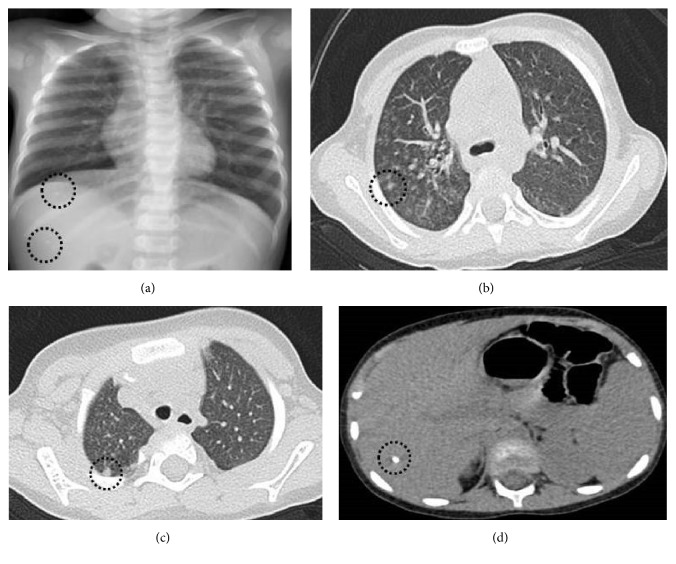
(a) Chest X-ray: bilateral infiltrations, microcalcifications in chest and abdomen. (b) and (c) Thorax CT: multiple, parenchymal nodules in both lungs, the biggest one 5 mm in diameter. (d) Thorax CT, the slices passing liver: a calcific nodule (At 26 months old).

She had failure to thrive (height and weight under the third percentile), oral Candidiasis, and bronchitis at admission. Complete blood count and biochemistry were normal. C-reactive protein was 1.7 mg/dL (normal: <0.3 mg/dL), and erythrocyte sedimentation rate was 10 mm/hour (normal: <20 mm/hour). Chest X-ray revealed bilateral microcalcifications and chronic lung disease findings. There were bilateral multiple parenchymal nodules, the biggest one being 5 mm, in high-resolution CT (Figure 1). An 8 mm calcific nodular lesion of the liver was reported and interpreted as a granuloma in the abdominal US.

*Candida albicans* was isolated from oral mucosa swab. Blood* Aspergillus* antigen was negative. Rhinovirus antigen was detected in a nasal swab. CMV and EBV DNA (1966 IU/ml and 7600 IU/ml, resp., by PCR) were present in blood and successfully treated with ganciclovir, significantly lowering the titers. However, these tended to rise again with pneumonia or gastroenteritis attacks or upon temporary withdrawal of the drug, suggesting chronicity. As oral fluconazole was not effective for Candidiasis, parenteral caspofungin was initiated with good response.* E. coli* grew in urine culture which was responsive to proper antibacterial medication. Tuberculin skin test was negative; interferon-gamma release assay (IGRA) was positive.

Mucopurulent secretions and mucous plaques on airways with normal anatomical structure were observed by bronchoscopy. Bronchoalveolar lavage fluid had benign cytology, and there was no evidence of bacteria, fungi, parasites, or mycobacteria. Repeated IGRA was found to be negative, and isoniazid (INH) prophylaxis was preferred instead of multidrug tuberculosis treatment. It was discontinued due to increased liver enzymes after the second month.

Investigations for persistent diarrhea showed negative results for viruses, parasites, and bacteria. Recurrent vomiting was also a problem in the patient. Upper gastrointestinal barium scan and pH monitorization did not show gastrointestinal reflux. The endoscopic evaluation revealed inflammatory findings in esophagus and bulbus with a loose lower esophageal sphincter. There were no candidal plaques, probably because she was under antifungal treatment at the time of examination. Pathological studies showed eosinophilic esophagitis and fibrosis.

Vaccine responses to Hepatitis B, Rubella, and Mumps were positive. Autoantibodies (antinuclear antibody, anti-gliadin antibodies, anti-thyroid peroxidase, and anti-thyroglobulin antibodies) and direct Coombs tests were negative, and thyroid hormone levels were normal.

First line immunological workup revealed hypergammaglobulinemia with normal complement and IgE levels. Percentages and numbers of lymphocyte subgroups were normal compared to age-related healthy controls [4–6] (Table 1). The quantitative determination of oxidative burst, the foxp3 expression on CD4+CD25+ T cells, and in vitro T-cell proliferation response to mitogens were normal.

She had chronic recurring infections with various pathogens including fungi, viruses, and bacteria. She had hypergammaglobulinemia, persistent oral* Candidiasis*, persistent granulomas, and nodules in the liver and the lungs. She was unresponsive to oral treatment and required parenteral treatments with frequent, over one-week hospitalizations warranting extensive workout for PID. Type and course of the infections with substantial organ damage suggested the presence of a severe combined immune deficiency, Mendelian susceptibility to mycobacterial diseases (MSMD), or CMC as preliminary diagnoses.

**Figure 2:**
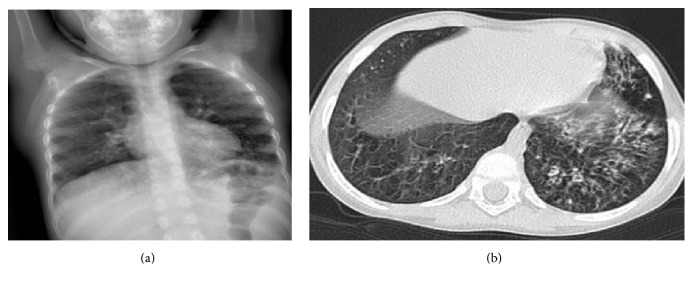
(a) Chest X-ray: bilateral infiltrations, bronchial thickenings, and air trapping. (b) Thorax CT: bronchiectasis in the left upper lobe and lower lobe, peribronchial thickenings, mucous plaques, and air trapping in both lungs (At four years of age).

Regular intravenous immunoglobulin (IVIG) replacement was commenced when she was three years old, as a supportive measure to frequent hospitalizations for pneumonia and diarrhea attacks. Routine prophylactic antibacterial and antifungal therapies besides ganciclovir were continued. Despite these measures, she developed bronchiectasis at the age of four (Figure 2).

**Figure 3:**
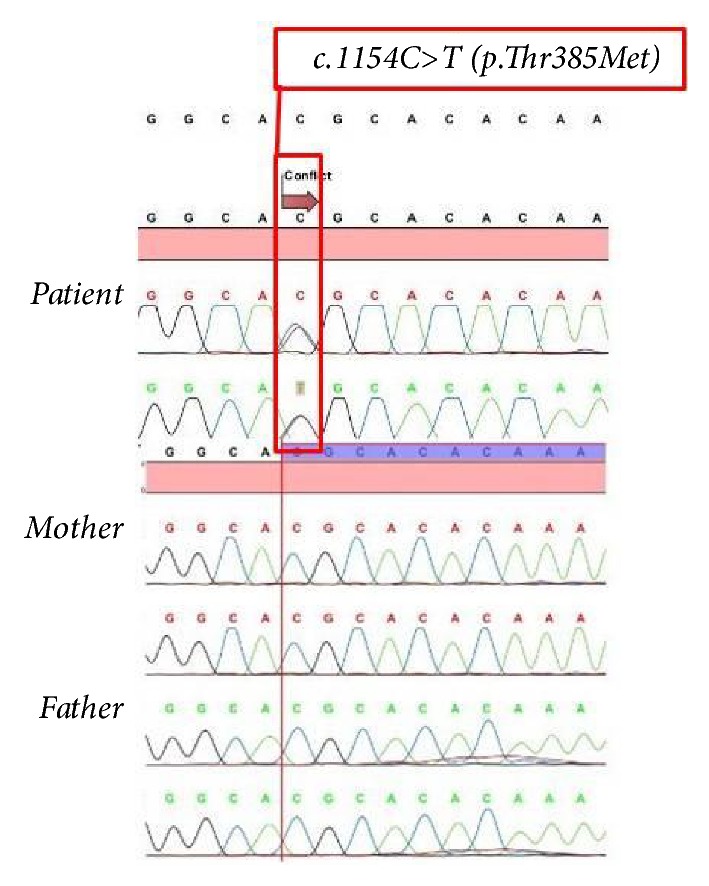
Identification of* de novo* autosomal dominant* STAT1* mutation in the patient.

Molecular genetic analyses of IL12R/IFN gamma and Th17-IL17 pathways were planned and the former was found to be negative. The investigation of the Th17/IL17 pathway revealed a heterozygous gain-of-function,* de novo* mutation in* STAT1* gene, which was described recently (c.1154C>T, p.Thr385Met, T385M). Parents were wild-type for the gene (Figure 3). HSCT was planned because of ongoing infections despite adequate supportive measures. There was no matched related donor. Thus, an unrelated donor search was initiated.

## 3. Discussion

Early-onset infections with diverse microorganisms, accompanying CMC, warrant search for combined immunodeficiency. Our case had normal lymphocyte subgroup distribution and normal in vitro T-cell proliferation response to mitogens. The granulomas in the lungs and the liver, hypergammaglobulinemia, and positive IGRA compatible with a mycobacterial infection and persistent candidal infection directed us to investigate IL-12/IFN gamma and Th17-IL17 pathways. However, a positive mycobacterial evidence could not be confirmed; she neither developed new lesions nor disseminated disease after cessation of INH which ruled out the possibility of MSMD. Conversely, the granulomas and nodules disappeared in follow-up with continuous parenteral antifungal treatment.

Impaired IL-17 immunity is the underlying cause of CMC. T helper 17 (Th17) cells and their effector cytokines IL-17 (IL-17A, IL-17F) and IL-22 have critical functions for candidal host defense via epithelial cells [3, 7, 8]. IL-17A and IL-17F bind to interleukin-17 receptor A (IL-17RA) and IL-17RC, respectively, in various tissues [8]. In this IL-17 signaling pathway, homozygous IL17RA, IL17RC, ACT 1, and heterozygous IL17F mutations were found to be causative in some CMCD patients [1, 2, 9]. Many patients in this group could not be identified genetically until GOF heterozygous missense mutations in the* STAT1* molecule were defined in this decade [10, 11]. After that, GOF* STAT1* mutations were found to be responsible for more than half of CMCD patients [1, 2].

STAT molecules are signal transducers for regulation of transcription in various cell types. After stimulation with cytokines (e.g., IFN-*γ*), STAT1 molecules are phosphorylated, dimerized, and translocated into the nucleus for gene expression and then dephosphorylated and released to the cytoplasm [12]. Proinflammatory cytokines, such as IL-6 and IL-21, activate STAT3, subsequently inducing transcription of IL-17 and IL-22 in T cells, transforming them into Th17 cells. Some interferons and cytokines (IFN-*α*/*β*, IFN-*γ*, IFN-*λ* and IL-27) activate STAT1, inhibiting transcription of IL-6 and IL-21 [12, 13]. In GOF* STAT1* mutations, dephosphorylation is defective causing prolonged phosphorylation and gain-of-function for STAT1-dependent cytokines inhibiting STAT3-mediated Th17 cell differentiation [1, 2, 10, 13].

To date, more than thirty GOF* STAT1* mutations were identified. Early reported mutations were all in the coiled-coil domain (CCD) of* STAT1* gene [1, 2, 10, 11]. Firstly discovered mutation in DNA binding domain (DBD) was a T385M mutation (as in our case) in two unrelated patients from Japan. The investigators showed STAT1 hyperphosphorylation (due to impaired dephosphorylation) in response to IFN-*γ*, IFN-*α*, and IL-27 stimulation and defective Th17 cell differentiation as in CCD mutations. Both patients suffered from recurrent oral Candidiasis and lower and upper respiratory tract infections that led to bronchiectasis in childhood period as in our case. Autoimmune diseases developed in the late childhood period in both of them; our patient did not have any autoimmune manifestation for the moment [14]. Enhanced IFN-*α*/*β* response may contribute to autoimmunity which is a late and variable finding [2, 10].

In an article evaluating nine patients with* STAT1* GOF mutations, including one patient carrying a T385M mutation, functional studies confirmed gain of phosphorylation and impaired Th17 response to Candida. Most obvious findings in this patient were early esophageal Candidiasis causing strictures and recurrent HSV infections [15]. Although macroscopic esophageal candidal involvement was not reported in our case, biopsy evaluation as eosinophilic esophagitis and fibrosis might be a hint in that direction.

Phenotypic presentation in* STAT1* GOF mutations may be heterogeneous and may not be directly related to the specific mutation. Diverse clinical features of the recently reported patients support this observation [1, 2].

Uzel et al. demonstrated* STAT1* GOF mutations in five patients with IPEX-like features (autoimmunity and enteropathy) and accompanying CMC phenotype. Two patients with the same mutation (T385M) had different clinical presentation: one had CMC and enteropathy as the prominent features starting in infancy with recurrent respiratory infections, bronchiectasis, recurrent viral infections, cerebral aneurysms, and autoimmunity (Type 1 diabetes mellitus). The other patient had very mild CMC and recurrent upper respiratory infections presenting in early childhood. Short stature was a common feature and most likely related to enteropathy suggesting the IPEX-like disease. Four of the five patients showed eosinophilic/lymphocytic enteritis [16]. Our case had recurrent diarrhea without chronicity. Her failure to thrive may be due to mild enteropathy or malnutrition secondary to frequent infections.

Patients with* STAT1* GOF mutations have various infections with bacteria, viruses, and mycobacteria. Infrequent invasive fungal infections are also seen [1, 2]. Five patients, each with different* STAT1* GOF mutations (including T385M), were reported to have disseminated infections with intracellular dimorphic fungi such as* Coccidioides immitis* and* Histoplasma capsulatum* [17]. Loss of function mutations (complete/partial recessive or dominant) in* STAT1* gene cause increased susceptibility to viral, bacterial, and mycobacterial infections varying in severity, and the underlying mechanism is impaired IFN-*α*/*β*, IFN-*γ* responses to these intracellular microorganisms [2, 12]. Conversely, IFN-*γ* and IFN-*α* induced STAT1 phosphorylation is enhanced in GOF* STAT1* mutations [14–16]. The impaired response to IFN restimulation due to prolonged phosphorylation which leads to a loss of function presents a possible explanation for increased susceptibility to viral, mycobacterial, and dimorphic fungal infections [17].

Our case presented with a combined immunodeficiency (CID) phenotype resembling several cases reported before [18, 19]. Two patients (one with T385M mutation) presented in early infancy with Candidiasis, CMV infection, and chronic lung findings. One of them also developed a mycobacterial infection. Despite CID findings, both patients had normal lymphocyte subgroup distribution except reversed CD4/CD8 ratio. One of them had low and the other normal proliferation response to mitogens [19]. Some* STAT1* GOF mutated patients developed T and B cell lymphopenia, hypogammaglobulinemia, and loss of memory B cells as with age. This progressive loss of immunologic functions may be another possible reason for newly acquired infections in time [16]. On the other hand, most patients (% 70–80) had normal lymphocyte ratios, immunoglobulin levels, and T-cell functions despite the high rate of infections [2]. Our case already had CMV and EBV viremia at admission while she had normal lymphocyte distribution and function. Namely, both cellular defects and abnormal regulation of IFN-*α*/*β*, IFN-*γ* responses are likely mechanisms underlying noncandidal infection susceptibility in* STAT1* GOF mutations.

Management of patients with* STAT1* GOF mutations should target prevention and treatment of infections. Prophylactic antimicrobials and IVIG are routinely used for this purpose [2, 16]. Since these mutations are just recently defined, HSCT is reported in a small number of patients and accepted as a radical treatment modality spared for patients with severe morbidity [2, 18–20]. Most patients are followed with various diagnoses as CID, IPEX-like syndrome and even undergo HSCT before identification of the molecular defect. Unfortunately, more than half of the reported patients died after transplantation due to multiple complications, most probably related to the late decision or poor selection of patients for HSCT [2, 18–20].

## 4. Conclusion

Careful consideration to the possibility of STAT1 GOF mutations should be given at the time of CMC diagnosis since they are reported to be causative in more than half of CMC patients. These patients may exhibit quite different phenotypes. New infections, autoimmune diseases, even malignancies, and aneurysms may emerge gradually with age. The fact that CMC is heterogeneous, progressive, and unpredictable in its course should alert physicians to recognize early stem cell transplantation decision as a feasible treatment option to avoid severe morbidity and mortality.

## Figures and Tables

**Table 1:** Serum immunoglobulin, complement levels, lymphocyte subgroups as ratios, and absolute cell numbers with age-related reference values [4–6].

	Patient	Reference values (mean ± SD)	Min–max
IgG (mg/dL)	1320	822.3 ± 208.4	430–1290
IgA (mg/dL)	98.9	53.5 ± 26.8	23–130
IgM (mg/dL)	149	92.5 ± 33.9	36–199
IgE (IU/mL)	0.9	<100	2–199
C3 (mg/dL)	171	120 ± 45	81–171
C4 (mg/dL)	28.3	22 ± 13	9–36
CD3+ T cells (%) (cells/mm^3^)	71 2620	70.0 ± 7.18 3220 ± 1180	48.2–81.4 506–7267
CD19+ B cells (%) (cells/mm^3^)	23 849	16.5 ± 5.70 739 ± 329	6.7–30.4 242–1459
CD3+CD4+ Th cells (%) (cells/mm^3^)	40 1476	40.3 ± 7.27 1314 ± 542	23.2–59.5 118–3245
CD3+CD8+ Tc cells (%) (cells/mm^3^)	28 1033	24.2 ± 5.48 803 ± 417	15.2–39 108–2367
CD3−CD1656+ NK cells (%) (cells/mm^3^)	5 185	11.2 ± 4.85 509 ± 295	3.4–26.4 143–1599
CD3+HLA-DR+ active T cells (%) (cells/mm^3^)	18 664	7.84 ± 3.7 375 ± 235	2.1–16.2 22–954
